# Autoregressive transitional ordinal model to test for treatment effect in neurological trials with complex endpoints

**DOI:** 10.1186/s12874-016-0251-y

**Published:** 2016-11-08

**Authors:** Lorenzo G. Tanadini, John D. Steeves, Armin Curt, Torsten Hothorn

**Affiliations:** 1 Department of Biostatistics; Epidemiology, Biostatistics and Prevention Institute, University of Zurich, Hirschengraben 84, Zurich, 8001 Switzerland; 2 ICORD, University of British Columbia and Vancouver Coastal Health, Vancouver, Canada; 3 Spinal Cord Injury Center, Balgrist University Hospital, Zurich, Switzerland

**Keywords:** Upper extremity motor scores, Summed overall score, Multivariate ordinal endpoints, Proportional odds model, Statistical power, Spinal cord injury, Sygen®; trial, Rasch models, Latent variable models

## Abstract

**Background:**

A number of potential therapeutic approaches for neurological disorders have failed to provide convincing evidence of efficacy, prompting pharmaceutical and health companies to discontinue their involvement in drug development. Limitations in the statistical analysis of complex endpoints have very likely had a negative impact on the translational process.

**Methods:**

We propose a transitional ordinal model with an autoregressive component to overcome previous limitations in the analysis of Upper Extremity Motor Scores, a relevant endpoint in the field of Spinal Cord Injury. Statistical power and clinical interpretation of estimated treatment effects of the proposed model were compared to routinely employed approaches in a large simulation study of two-arm randomized clinical trials. A revisitation of a key historical trial provides further comparison between the different analysis approaches.

**Results:**

The proposed model outperformed all other approaches in virtually all simulation settings, achieving on average 14 % higher statistical power than the respective second-best performing approach (range: -1 %, +34 %). Only the transitional model allows treatment effect estimates to be interpreted as conditional odds ratios, providing clear interpretation and visualization.

**Conclusion:**

The proposed model takes into account the complex ordinal nature of the endpoint under investigation and explicitly accounts for relevant prognostic factors such as lesion level and baseline information. Superior statistical power, combined with clear clinical interpretation of estimated treatment effects and widespread availability in commercial software, are strong arguments for clinicians and trial scientists to adopt, and further extend, the proposed approach.

**Electronic supplementary material:**

The online version of this article (doi:10.1186/s12874-016-0251-y) contains supplementary material, which is available to authorized users.

## Background

Neurological research is responsible for the investigation of many devastating disorders such as stroke, Alzheimer’s and Parkinson’s diseases. In terms of health costs, brain-related disorders are a greater socio-economic burden than cancer, cardiovascular diseases and diabetes combined [1], with yearly costs for the European society estimated at almost 400 billion € [2].

Despite several therapeutic approaches [3–6] based on recent discoveries of cellular and molecular processes of degeneration, but also spontaneous regeneration following injury, pharmaceutical and health companies have been withdrawing from neuroscience, as a number of trials intended to show efficacy of treatments for neurological disorders failed [7]. In the field of Spinal Cord Injury (SCI), four decades after the first pharmacological treatment of acute injuries [8], the promises of preclinical discoveries have yet to be translated into a standard treatment [9].

To streamline the translational process, the International Campaign for Cures of Spinal Cord Injury Paralysis (ICCP) appointed in 2007 an international panel with the task to reviewing strengths and weaknesses of clinical trials in spinal cord injury. Their recommendations for the planning and conduction of future trials were condensed in a series of publications [10–13], which strongly influenced the conception of clinical trials thereafter [14].

Nonetheless, the ICCP reviews [10–13] did not solicit the application of the most appropriate and recent statistical techniques available for the analysis of complex SCI trial endpoints, and many clinical trials failed to do so too [15–19].

In fact, virtually all routinely performed clinical assessments in spinal cord injury are measured on ordinal scales, which are characterized by an arbitrary numerical score establishing a ranking of observations. The difference between two following ranks is by no means bound to be equivalent across the range of the scale, preventing standard operations such as addition, and making the use of statistical methods developed for continuous endpoints inappropriate. Despite this, clinical trials designed and powered for a primary ordinal endpoint often resorted to adding several ordinal endpoints to form a single overall summed score, which is in some cases subsequently collapsed to a binary outcome [15–19]. These approaches have been shown to be inappropriate in a number of aspects [20], and practical consequences such as biased parameter estimates, misleading associations and loss of power are some of the known consequences of assuming metric properties for ordinal endpoints [21–23].

In this study, we propose for the first time in SCI a transitional ordinal model with an autoregressive component for testing for treatment effect on a multivariate ordinal endpoint such as the Upper Extremity Motor Scores (UEMS), while comparing it to current analysis approaches in terms of statistical power and clinical interpretation of treatment effect estimates.

## Methods

The objective was to propose a new approach to the analysis of complex ordinal endpoints in neurological clinical trials, and provide statistical power comparisons of procedures for treatment effect testing. Two-armed Randomized Clinical Trials (RCT) with specific levels of experimental conditions were generated and analysed. Current approaches to the analysis of multivariate ordinal endpoints such as the Upper Extremity Motor Scores (UEMS) were compared to the proposed autoregressive transitional ordinal model. The proposed approach models the transition, e.g. the change in UEMS distribution, from trial baseline to trial end. The autoregressive term of the model describes the anatomical structure of the spinal cord by postulating a direct dependency between contiguous segments.

### Data source and trial endpoint

The data utilized in this study was extracted from the European Multicenter Study about Spinal Cord Injury (EMSCI, ClinicalTrials.gov Identifier: NCT01571531, www.emsci.org). EMSCI tracks the functional and neurological recovery of patients during the first year after spinal cord injury in a highly standardized manner. All patients gave written informed consent. The ethical committee of the Canton of Zurich, Switzerland, has previously approved the EMSCI project, upon which this project is based, and the approval is also valid for any statistical analysis/re-analysis.

To reflect the time frame of a possible future clinical trial, we considered baseline (within 2 weeks after injury, *t*=1) and one follow-up (6 months after injury, *t*=2) examination. For this simulation study, we extracted and utilized records of N=405 patients with a Motor Level (ML) defined between spinal segments C5-T1 (see Additional file 1 for details) and with available baseline information.

The trial endpoint considered is the Upper Extremity Motor Scores. UEMS represents a subset of the International Standards for Neurological Classification of Spinal Cord Injury (ISNCSCI) [24] and describes the muscle contraction force for 10 key muscles on the arms and hands (5 on each body side), each one being rated on a 6-point ordinal scale (0: total paralysis, through 5: active movement against full resistance, see Additional file 1 for details). Accordingly, *Y*_*i,m,t*_ is the muscle contraction score for patient *i* (*i*=1,…,*n*) and key muscle *m* (*m*=1,…,10) measured at time point *t* (*t*=1,2). Each key muscle *Y*_*i,m,t*_ is therefore an ordinal variable with *k*=6 levels 0<1<…<5, and UEMS is a multivariate ordinal endpoint. The chosen endpoint is particularly relevant in SCI. A change in total UEMS over trial period has been employed repeatedly in clinical trials [15, 19] and has been suggested to correlate with changes in activities of daily living that rely on recovery of upper extremity function [25].

### RCT simulation

An autoregressive transitional ordinal model of the form:

1 $$  {}\begin{aligned} logit \left[ P(y_{i,m,2} \leq k)\right] &= \alpha_{j} + \beta_{\text{lev}}\hspace{1mm}x_{\text{lev},i,m,1} + \beta_{\text{base}} \hspace{1mm}y_{\text{base},i,m,1}\\ & \quad + \beta_{\text{auto}} \hspace{1mm}y_{\text{auto},i,m-1,2} \end{aligned}  $$

was fitted on the EMSCI data. *α*_*j*_ are the *k*−1=5 intercept parameters, *x*_lev_ is a 10-level nominal factor denoting the combination of Motor Level and the distance from the Motor Level to the key muscle *m* being analysed, expressed as number of key muscles along the spine (reference: motor level: cervical C5, distance: -1 (first muscle below the level)), *y*_base,*i,m*,1_ is the ordered factor for baseline motor score of key muscle *m*, and *y*_auto,*i,m*−1,2_ is the ordered factor for motor score of the key muscle just above the one being analysed at *t*=2. The autoregressive term of the model describes the anatomical structure of the spinal cord, and postulates that the motor score of a given key muscle depends on the Motor Score of the key muscle just rostral to it. As a consequence, the observed pattern of lower motor scores with increasing distance from the ML is reproduced. In accordance with the above description, Eq. 1 simulated and analysed only key muscle score below the Motor Level. Motor scores *y*_*i,m*,2_ for key muscles at ML were multinomially sampled from corresponding observed EMSCI frequencies at Motor Level, while motor scores *y*_*i,m*,2_ for key muscles above the ML were given the maximal score.

The parameter estimates recovered from the model specified in Eq. 1 describe the spontaneous neurological recovery for patients under standard of care and were subsequently used to simulate participants in the control arm of the trial. From the EMSCI data we also computed the observed frequencies of Motor Level combinations for the left and right body side at baseline. Given that patients having both left and right ML at the lowest UEMS key muscles T1 are very rare (3 % in our EMSCI sample) and do not contribute to the analysis (no key muscles in the UEMS below the ML), they were not included into the simulation.

Equation 1 models the spontaneous neurological recovery for patients under standard of care. We introduced an additional parameter *β*_trt_ representing a postulated treatment effect, leading to an autoregressive transitional ordinal model of the form:

2 $$  {}\begin{aligned} logit \left[ P(y_{i,m,2} \leq k)\right] &= \alpha_{j} + \beta_{\text{lev}}\hspace{1mm}x_{\text{lev},i,m,1} + \beta_{\text{base}} \, y_{\text{base},i,m,1}\\ & \quad + \beta_{\text{auto}} \hspace{1mm}y_{\text{auto},i,m-1,2} + \beta_{\text{trt}}\hspace{1mm}x_{\text{trt},i,1} \end{aligned}  $$

As previously defined, *α*_*j*_ are the *k*−1=5 intercept parameters, *x*_*lev*_ is a 10-level nominal factor denoting the combination of Motor Level and the distance from the Motor Level to the key muscle *m* being analysed, expressed as number of key muscles along the spine (reference: Motor Level: C5, distance: -1), *y*_base,*i,m*,1_ is the ordered factor for baseline motor score of key muscle *m*, *y*_auto,*i,m*−1,2_ is ordered factor for motor score of the key muscle just above the one being analysed at *t*=2, and *x*_trt_ is an indicator for treatment arm with placebo as reference.

The autoregressive term of the model describes the anatomical structure of the spinal cord, and postulates that the motor score of a given key muscle depends on the motor score of the key muscle just rostral to it. As a consequence, the observed pattern of lower motor scores with increasing distance from the ML is reproduced. Besides the postulated treatment effect *β*_trt_, which is set to different values depending on the simulation settings, all other parameters in Eq. 2 were kept equal to the estimates recovered by fitting Eq. 1 to the EMSCI data.

We thus simulated randomized clinical trials with two treatment arms and specific levels of experimental conditions. To cover possible SCI early phase as well as phase III settings, we generated total trial sample sizes of 50, 75, 100, 125, 150, 175, 200 participants. To our knowledge, there is to date no publication on the magnitude of possible treatment effects for UEMS which could have guided us in defining more tailored scenarios. We therefore postulated a rather wide range of six possible treatment effects (from no treatment effect (*β*_trt_=0.0= log(1)) to strong treatment effect (*β*_trt_=0.4055= log(1.5)) in 0.1 steps). A total of 42 scenarios resulted from simulating all possible combinations of the 7 trial sample sizes and 6 possible treatment effects considered. Being a proportional odds model, the exponentiated *β*_trt_ can be interpreted as conditional Odds Ratio (OR) between trial arms, meaning that, conditional on all other prognostic factors being equal, it specifies the ratio of the odds for a key muscle to achieve a motor score of less than or equal to *k* in the treatment arm divided by the same odds in the control arm. OR is a statistically sensible and clinical widely accepted way of quantifying effects of categorical variables.

The 42 trial scenarios resulting from all combinations of 7 trial sample sizes and 6 possible treatment effects were simulated in the following way:

1. Right and left Motor Levels for the hypothesized number of trial participants were drawn from a multinomial distribution with category probabilities set to the corresponding observed EMSCI frequencies.
2. Baseline UEMS for each trial participant were sampled with replacement from all EMSCI patients having the same left-right ML constellation.
3. Each simulated participant was randomly allocated to either the control or the treatment arm with a 1:1 allocation scheme.
4. UEMS at six months for the key muscle at ML were drawn from a multinomial distribution with category probabilities set to the corresponding observed EMSCI frequencies.
5. UEMS at six months below the ML were simulated using the previously fitted model for spontaneous recovery (Eq. 1) for participants in the control arm, and the same model with the addition of a postulated treatment effect (Eq. 2) for participants in the treatment arm of the trial.
6. Each one of the 42 trial scenarios was replicated 1000 times.
7. A battery of 6 different tests for treatment effect (see below “Endpoint analysis approaches” Section) were applied to each simulated trial.
8. The statistical power =*P*(reject *H*_0_|*H*_1_ is true) was estimated as the fraction of significant tests for treatment effect at the nominal level 0.05 among the 1000 replications.

### Endpoint analysis approaches

In neurology in general, and SCI in particular, very common approaches to the analysis of UEMS or similar endpoints are as the total sum of all motor scores $Y^{*}_{i,2} = \sum _{m=1}^{10} Y_{i,m,2}$ or as difference between two time points $Y^{**}_{i} = \sum _{m=1}^{10} Y_{i,m,2} - Y_{i,m,1}$. Accordingly, treatment effect for UEMS was tested with:

- **t-test:** t-test for $ Y^{*}_{i,2}$, comparing mean total UEMS in the two treatment groups.
- **t-test delta:** t-test for $ Y^{**}_{i}$, comparing the mean difference in total UEMS from baseline to the end of the trial between the two treatment groups.
- **ANCOVA:** Analysis of covariance for $Y^{*}_{i,2}$, comparing mean total UEMS in the two treatment groups with baseline total UEMS $ Y^{*}_{i,1}$ as controlling continuous variable.

Even though not commonly done in SCI, we considered necessary that the Motor Level should be incorporated into the analysis of motor function. In fact, its importance has been reported before [26, 27]. We therefore applied a conditional test of independence between outcome and treatment arm which was stratified according to the Motor Level of each trial participant. We predicted that this approach would perform better than the previous, not stratified ones, and explored the possibility to utilise them as “ad hoc” approach for the analysis of UEMS. Accordingly, treatment effect for UEMS was tested with:

- **i-test:** stratified independence test for $Y^{*}_{i,2}$, comparing total UEMS in the two treatment groups.
- **i-test delta:** stratified independence test for $Y^{**}_{i}$, comparing the difference in total UEMS from baseline to the end of the trial between the two treatment groups.

Both tests are implemented in the R add-on package **coin** [28, 29].

The last approach for the analysis of UEMS in a RCT is a model that takes into account the ordinal nature of each key muscle and explicitly incorporates baseline UEMS as well as ML into the analysis:

- **transitional:** transitional ordinal model for *Y*_*i,m*,2_ of the form specified in Eq. 2, comparing the shift in motor score probabilities associated with treatment.

The proposed model is a proportional odds model with an autoregressive component. The latter takes into account the spatial orientation of the key muscles along the spinal cord by postulating a direct dependency of adjacent spinal segments. As a consequence, the observed pattern of lower Motor Scores with increasing distance from the ML is reproduced. This model was fitted using function polr from the R add-on package **MASS** [30, 31].

The parameter *β*_trt_, which quantifies the treatment effect on the link scale, is the focus of the proposed model. Its significance testing was based on a permutation test [32, 33], where the distribution of the test statistics under *H*_0_ (no treatment effect) was based on refitting the same model 1000 times after randomly rearranging the labels for arm allocation. This type of statistical significance test does not rely on any distributional assumption. In addition, by permuting trial arm allocation at participant level, we accounted for the hierarchical structure of the data analysed, where multiple key muscles are measured on the same participant. All computations were performed in the R system for statistical computing [34], version 3.1.3. The R code implementing the simulation study is available online (doi: http://dx.doi.org/10.5281/zenodo.47600).

### Revisiting a key SCI trial

As a practical application, we analysed a subset of the data collected during a past clinical trial. The Sygen ®;trial recruited *N* =760 SCI participants in 28 centres in North-America in a 5-year period between 1992 and 1997 [17, 35, 36]. Sygen ®;is a naturally occurring compound in cell membranes which has been associated with neuroprotective and regenerative effects in a number of experimental models and early-phase human trials. The trial is an example where a promising therapeutic approach was finally abandoned, as no significant treatment effect could be assessed on the primary endpoint despite a considerable final sample size (*N* =760). The primary endpoint assessed the overall neurological status of a patient and was defined as a dichotomization derived from an ordinal scale (see [36] for the exact definition). The primary endpoint was analysed by means of logistic regression. Several ancillary analyses were performed and mostly preferred the treatment arm, even though the differences were not always statistically significant. To our knowledge, no analysis performed at the level of motor scores of the upper extremity key muscles UEMS as reported here have been published.

We revisited the trial by testing for treatment effect on the UEMS with all six approaches outlined before (see “Endpoint analysis approaches” Section). The proposed autoregressive transitional ordinal model (Eq. 2) can be easily fitted as proportional odds model to the segment-wise UEMS data in the long format. The autoregressive component *y*_*i,m*−1,2_ can be incorporated by shifting the six-month, muscle-wise UEMS entries so as to be aligned to the key muscle *y*_*i,m*,2_ just caudal to them.

To reflect our simulation study, we selected participants with a ML between C5 - C8 (T1 were discarded, because there is no key muscle caudal to the ML on the UEMS), and considered only patients treated with a low dosage (the original trial had two treatment doses, the higher of which was abandoned during the study). After patients selection, we analysed a finale sample of *N* =284 participants, 127 (45 %) of which in the control arm. This analysis is intended to give an example of the application of the proposed transitional ordinal model, but is not intended and should not be taken as a definitive conclusion about the value or outcome of the trial. Given the strongly selected patients sample utilised, the different endpoint analysed and the different scope of our analysis, generalizations of this type cannot be drawn.

## Results

### RCT simulation

**Fig. 1:**
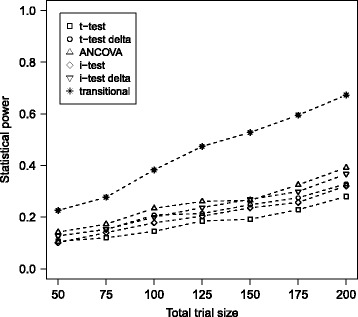
Comparison of statistical power for the median treatment effect. The statistical power of all six approaches for treatment effect testing are plotted against total trial size (1:1 randomization) for the median simulated treatment effect *β*_trt_=0.2624= log(1.3)

**Fig. 2:**
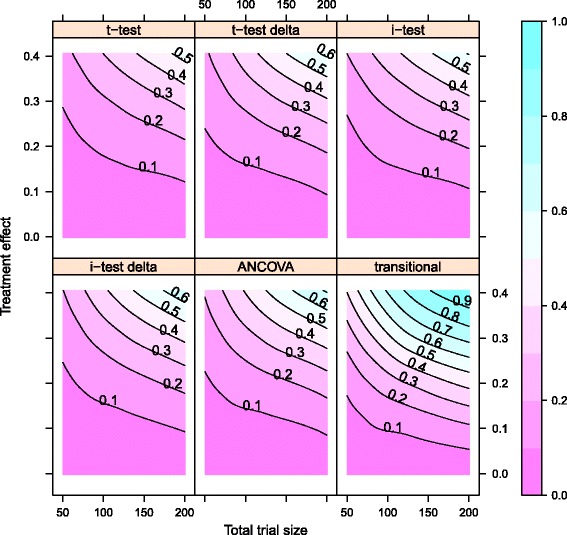
Contour plots of statistical power for all simulation settings. The statistical power of all testing approaches is represented using loess smooth approximation. Contour curves visualize combinations of trial size and treatment effect with equivalent statistical power, which is reported as numerical value. The colour key differentiates regions of low statistical power (*violet*) from regions of high statistical power (*blue*)

For the purpose of this study, we simulated 1000 times each one of the 42 different combinations of trials size and postulated treatment effect. Statistical power, which is defined as the probability of rejecting the *H*_0_ of no treatment when there is in fact a treatment effect, was estimated as the fraction of this 1000 iterations where the test for treatment effect resulted significant at the 0.05 level. Table 1 reports the statistical power of all treatment testing approaches for all simulated settings. Figure 1 shows the statistical power of all six approaches for the intermediate treatment effect simulated. Figure 2 displays graphically the statistical power of all treatment testing approaches for all simulated settings. The nominal level 0.05 was maintained by all approaches when no treatment effect was introduced in the simulation, making further comparisons between different approaches straightforward.

**Table 1:** Statistical power for all simulation settings. Point estimates, as well as Wilson confidence intervals are reported for all analysis approaches

Size	Treatment	OR	T-test	CI lower	CI upper	T-test delta	CI lower	CI upper	I-test	CI lower	CI upper	I-test delta	CI lower	CI upper	ANCOVA	CI lower	CI upper	Transitional	CI lower	CI upper
50	0.0000	1.0	0.053	0.041	0.069	0.052	0.040	0.068	0.051	0.039	0.066	0.042	0.031	0.056	0.046	0.035	0.061	0.050	0.038	0.065
75	0.0000	1.0	0.048	0.036	0.063	0.050	0.038	0.065	0.052	0.040	0.068	0.051	0.039	0.066	0.053	0.041	0.069	0.052	0.040	0.068
100	0.0000	1.0	0.047	0.036	0.062	0.046	0.035	0.061	0.054	0.042	0.070	0.046	0.035	0.061	0.048	0.036	0.063	0.045	0.034	0.060
125	0.0000	1.0	0.049	0.037	0.064	0.052	0.040	0.068	0.040	0.030	0.054	0.056	0.043	0.072	0.056	0.043	0.072	0.057	0.044	0.073
150	0.0000	1.0	0.056	0.043	0.072	0.044	0.033	0.059	0.041	0.030	0.055	0.040	0.030	0.054	0.050	0.038	0.065	0.040	0.030	0.054
175	0.0000	1.0	0.050	0.038	0.065	0.050	0.038	0.065	0.043	0.032	0.057	0.053	0.041	0.069	0.042	0.031	0.056	0.047	0.036	0.062
200	0.0000	1.0	0.051	0.039	0.066	0.052	0.040	0.068	0.046	0.035	0.061	0.053	0.041	0.069	0.056	0.043	0.072	0.048	0.036	0.063
50	0.0953	1.1	0.057	0.044	0.073	0.060	0.047	0.076	0.063	0.050	0.080	0.052	0.040	0.068	0.062	0.049	0.079	0.049	0.037	0.064
75	0.0953	1.1	0.055	0.042	0.071	0.056	0.043	0.072	0.051	0.039	0.066	0.069	0.055	0.086	0.049	0.037	0.064	0.086	0.070	0.105
100	0.0953	1.1	0.057	0.044	0.073	0.071	0.057	0.089	0.061	0.048	0.078	0.071	0.057	0.089	0.071	0.057	0.089	0.106	0.088	0.127
125	0.0953	1.1	0.074	0.059	0.092	0.068	0.054	0.085	0.082	0.067	0.101	0.075	0.060	0.093	0.081	0.066	0.100	0.094	0.077	0.114
150	0.0953	1.1	0.063	0.050	0.080	0.070	0.056	0.088	0.062	0.049	0.079	0.075	0.060	0.093	0.078	0.063	0.096	0.116	0.098	0.137
175	0.0953	1.1	0.066	0.052	0.083	0.071	0.057	0.089	0.069	0.055	0.086	0.079	0.064	0.097	0.073	0.058	0.091	0.117	0.099	0.138
200	0.0953	1.1	0.072	0.058	0.090	0.101	0.084	0.121	0.080	0.065	0.098	0.092	0.076	0.112	0.099	0.082	0.119	0.135	0.115	0.158
50	0.1823	1.2	0.068	0.054	0.085	0.090	0.074	0.109	0.065	0.051	0.082	0.091	0.075	0.110	0.093	0.077	0.113	0.111	0.093	0.132
75	0.1823	1.2	0.096	0.079	0.116	0.095	0.078	0.115	0.106	0.088	0.127	0.100	0.083	0.120	0.107	0.089	0.128	0.164	0.142	0.188
100	0.1823	1.2	0.106	0.088	0.127	0.098	0.081	0.118	0.112	0.094	0.133	0.099	0.082	0.119	0.114	0.096	0.135	0.226	0.201	0.253
125	0.1823	1.2	0.115	0.097	0.136	0.127	0.108	0.149	0.135	0.115	0.158	0.132	0.112	0.154	0.145	0.125	0.168	0.261	0.235	0.289
150	0.1823	1.2	0.134	0.114	0.157	0.155	0.134	0.179	0.138	0.118	0.161	0.167	0.145	0.191	0.171	0.149	0.196	0.298	0.270	0.327
175	0.1823	1.2	0.134	0.114	0.157	0.161	0.140	0.185	0.166	0.144	0.190	0.177	0.155	0.202	0.182	0.159	0.207	0.331	0.303	0.361
200	0.1823	1.2	0.145	0.125	0.168	0.189	0.166	0.214	0.175	0.153	0.200	0.191	0.168	0.217	0.215	0.191	0.242	0.360	0.331	0.390
50	0.2624	1.3	0.106	0.088	0.127	0.128	0.109	0.150	0.101	0.084	0.121	0.127	0.108	0.149	0.142	0.122	0.165	0.226	0.201	0.253
75	0.2624	1.3	0.120	0.101	0.142	0.152	0.131	0.176	0.140	0.120	0.163	0.153	0.132	0.177	0.173	0.151	0.198	0.277	0.250	0.306
100	0.2624	1.3	0.145	0.125	0.168	0.208	0.184	0.234	0.178	0.156	0.203	0.200	0.176	0.226	0.234	0.209	0.261	0.383	0.353	0.414
125	0.2624	1.3	0.185	0.162	0.210	0.214	0.190	0.240	0.204	0.180	0.230	0.237	0.212	0.264	0.261	0.235	0.289	0.474	0.443	0.505
150	0.2624	1.3	0.192	0.169	0.218	0.248	0.222	0.276	0.236	0.211	0.263	0.269	0.242	0.297	0.265	0.239	0.293	0.528	0.497	0.559
175	0.2624	1.3	0.229	0.204	0.256	0.275	0.248	0.303	0.257	0.231	0.285	0.299	0.271	0.328	0.325	0.297	0.355	0.595	0.564	0.625
200	0.2624	1.3	0.280	0.253	0.309	0.329	0.301	0.359	0.321	0.293	0.351	0.367	0.338	0.397	0.392	0.362	0.423	0.673	0.643	0.701
50	0.3365	1.4	0.119	0.100	0.141	0.154	0.133	0.178	0.141	0.121	0.164	0.153	0.132	0.177	0.161	0.140	0.185	0.303	0.275	0.332
75	0.3365	1.4	0.184	0.161	0.209	0.195	0.172	0.221	0.212	0.188	0.238	0.209	0.185	0.235	0.240	0.215	0.267	0.410	0.380	0.441
100	0.3365	1.4	0.221	0.196	0.248	0.253	0.227	0.281	0.260	0.234	0.288	0.288	0.261	0.317	0.302	0.274	0.331	0.580	0.549	0.610
125	0.3365	1.4	0.290	0.263	0.319	0.314	0.286	0.343	0.308	0.280	0.337	0.339	0.310	0.369	0.396	0.366	0.427	0.692	0.663	0.720
150	0.3365	1.4	0.309	0.281	0.338	0.376	0.347	0.406	0.374	0.345	0.404	0.404	0.374	0.435	0.442	0.411	0.473	0.736	0.708	0.762
175	0.3365	1.4	0.329	0.301	0.359	0.399	0.369	0.430	0.396	0.366	0.427	0.434	0.404	0.465	0.463	0.432	0.494	0.800	0.774	0.824
200	0.3365	1.4	0.407	0.377	0.438	0.464	0.433	0.495	0.445	0.414	0.476	0.495	0.464	0.526	0.536	0.505	0.567	0.857	0.834	0.877
50	0.4055	1.5	0.162	0.140	0.186	0.178	0.156	0.203	0.196	0.173	0.222	0.190	0.167	0.215	0.210	0.186	0.236	0.392	0.362	0.423
75	0.4055	1.5	0.238	0.213	0.265	0.263	0.237	0.291	0.281	0.254	0.310	0.291	0.264	0.320	0.318	0.290	0.348	0.592	0.561	0.622
100	0.4055	1.5	0.302	0.274	0.331	0.354	0.325	0.384	0.366	0.337	0.396	0.390	0.360	0.421	0.392	0.362	0.423	0.737	0.709	0.763
125	0.4055	1.5	0.368	0.339	0.398	0.443	0.412	0.474	0.420	0.390	0.451	0.467	0.436	0.498	0.515	0.484	0.546	0.825	0.800	0.847
150	0.4055	1.5	0.397	0.367	0.428	0.509	0.478	0.540	0.467	0.436	0.498	0.546	0.515	0.577	0.583	0.552	0.613	0.891	0.870	0.909
175	0.4055	1.5	0.495	0.464	0.526	0.559	0.528	0.589	0.567	0.536	0.597	0.597	0.566	0.627	0.648	0.618	0.677	0.919	0.900	0.934
200	0.4055	1.5	0.530	0.499	0.561	0.616	0.585	0.646	0.598	0.567	0.628	0.669	0.639	0.697	0.706	0.677	0.733	0.967	0.954	0.976

For the smallest treatment effect *β*_trt_=0.0953= log(1.1), all six tests for treatment effect showed a low power, never exceeding *P*(reject *H*_0_|*H*_1_ is true) ≤0.135. The transitional ordinal model was nonetheless superior to all other approaches in virtually every trial size setting, its power point estimates averaging 2.3 % higher than the respective second best-performing approach.

Already at the next higher treatment effect simulated *β*_trt_=0.1823= log(1.2), the transitional ordinal model showed roughly twice as much power as the second-best performing approach, though it did not exceed *P*(reject *H*_0_|*H*_1_ is true) ≤0.36. This held for all simulation settings except the smallest sample size. Statistical power point estimates for the transitional ordinal model were on average 10.3 % higher than the respective second best-performing approach.

In the settings with median simulated treatment effect *β*_trt_=0.2624= log(1.3) shown in Fig. 1, the transitional ordinal model was superior for all trial sizes. Power point estimates for the proposed model were on average 19.4 % higher than the respective second best-performing approach, with this difference in performance increasing with increasing trial size.

With the simulated treatment effect of *β*_trt_=0.3365= log(1.4), the transitional ordinal model had superior statistical power of 26.3 % on average, compared to the respective second best-performing approach, with this difference increasing with increasing trial size.

For the largest simulated treatment effect of *β*_trt_=0.4055= log(1.5), the transitional ordinal model had an average superior statistical power of 27.9 %, compared to the respective second best-performing approach. The difference in performance increased strongly up to trial size *N* =100, but then declined with larger sizes.

Overall, despite a comparably poor performance of all approaches for small simulated treatment effects, a stable pattern in the ranking of performance emerged: the proposed transitional ordinal approach provided best power results in virtually all settings. ANCOVA was usually the second-best approach, closely followed by the independence test on the difference of UEMS from baseline $ Y^{**}_{i}$, the similarly performing t-test on the difference of UEMS from baseline $ Y^{**}_{i}$ and the independence test on the UEMS after six months $ Y^{*}_{i,2}$. The t-test on the UEMS after six months $ Y^{*}_{i,2}$ performed worst in almost all settings.

### Revisiting a key SCI trial

We analysed a subset of the data collected during the Sygen ®;trial [17, 35, 36]. To our knowledge, no analysis on this data has been performed at the level of motor scores of the upper extremity key muscles UEMS as reported here. The results of the six analysis approaches (see Endpoint analysis approaches section) are reported here:

- **t-test:** No significant difference in the estimated means $\widehat {\mu _{\text {ctrl}}}=30.370$ and $\widehat {\mu _{\text {trt}}}=30.170$ of UEMS at 6 months between trial arms: t(275)=0.130, *p*−value=0.896.
- **t-test delta:** No significant difference in the estimated mean change $\widehat {\mu _{\text {ctrl}}}=11.978$ and $\widehat {\mu _{\text {trt}}}=10.540$ of UEMS between trial arms: t(259)=1.239, *p*−value=0.216.
- **ANCOVA:** No significant difference in the estimated means of UEMS at 6 months between trial arms, controlling for baseline UEMS: $\widehat {\beta _{\text {trt}}}=-1.165$, *p*−value=0.307.
- **i-test:** No significant dependency between UEMS at 6 months and treatment arm: Z=0.553, *p*− value = 0.58.
- **i-test delta:** No significant dependency between change in UEMS and treatment arm: Z=1.525, *p*−value=0.127.
- **transitional:** No significant shift in motor score probabilities associated with treatment arm: $\widehat {\beta _{\text {trt}}}= -0.197$, *p*−value=0.207.

Summarizing, all six approached did not show significant results at the nominal level 0.05, but they all showed a tendency to less positive outcomes for patients in the treatment arm. This analysis is intended to give an example of the application for the proposed transitional ordinal model, but is not intended and should not be taken as a definitive conclusion about the value or outcome of the trial.

## Discussion

The aim of this simulation study was to compare several approaches of testing for treatment effect in two-armed RCT in a neurological setting. We therefore simulated clinical trials with cervical SCI participants with specific levels of experimental conditions and tested for treatment effect with six different approaches. Routinely employed analysis approaches not only rely on strong assumptions about the properties of the endpoints being analysed, but were also outperformed in virtually all settings by the the proposed autoregressive transitional ordinal model for the analysis of UEMS.

### Adding ordinal endpoints to form a single overall score is generally not valid

Common approaches to the analysis of UEMS and similar neurological endpoints are as the total sum of all motor scores $Y^{*}_{i,2} = \sum _{m=1}^{10} Y_{i,m,2}$ or as difference between two time points $Y^{**}_{i} = \sum _{m=1}^{10} Y_{i,m,2} - Y_{i,m,1}$.

Whether it is appropriate to combine a set of ordinal variables to generate a total score is usually not checked in neurology [37]. It should nonetheless be a requirement, as there are at least two strong assumptions related to the analysis of summed motor scores as a metric endpoint: unidimensionality and equal differences. Unidimensionality refers to the property of several scores to measure a single, common patient’s characteristic. While there is some preliminary evidence that unidimensionality holds for UEMS [38], the opposite was reported for both the Functional Independence Measure FIM [39], the Spinal Cord Independence Measure SCIM [40], a situation which is very likely to be found in functional endpoints and Patients Reported Outcomes PRO. Equal differences imply that a unit change in motor scores represent exactly the same clinical change, independently of where the change took place on the scale (e.g. a change from 0 to 1 is assumed to be of the same magnitude as a change from 3 to 4 in motor scores), or of which key muscle are considered (the previous example is assumed to hold even when the changes took place on different key muscles, say e.g. one proximal and one distal from the lesion level).

The widely used method of adding up several ordinal endpoints to form a single overall score is therefore generally not valid with regard to the two assumptions exemplified above, and has been repeatedly reported in neurological and related physical functioning settings [39–44]. From a practical point of view, biased parameter estimates, as well as misleading associations and loss of power are some of the known consequences of assuming metric property for ordinal endpoints [21–23]. There is therefore a compelling need to embrace statistical models specifically designed for the analysis of complex ordinal endpoints.

### RCT simulation

The proposed autoregressive transitional ordinal model is the first attempt in SCI to model and analyse a complex endpoint with a regression model which reflects its ordinal nature and takes into account important prognostic factors. The proposed model for the analysis of UEMS in cervical SCI patients outperformed all other approaches in virtually all settings. The sensibly lower statistical power achieved by commonly used approaches, in addition to their implicit assumptions, indicate that their use as default analysis methods in not justified.

Contrary to our expectations, a stratification of the t-test based on the Motor Level did not provide a discernible improvement in statistical power (Table 1). In fact, even though blocked independence tests showed a slightly higher power than their corresponding t-tests (Fig. 2), the gain in power was not such that their application as “ad hoc” solution resulted substantiated.

**Fig. 3:**
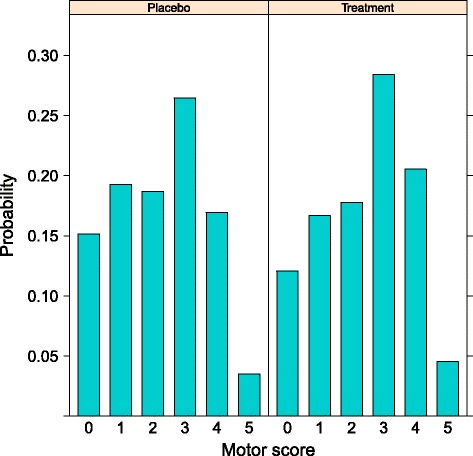
Visualization of median treatment effect *β*_trt_=0.2624=log(1.3). In contrast to all other analysis approaches, the transitional ordinal model allows to graphically represent shifts in motor score distributions for any constellation of relevant prognostic factors, permitting a much more detailed investigation of treatment effect. As illustrative example, represented is the distribution of motor score probabilities for participants in the control (*left* panel) and treatment arm (*right* panel). Lower scores became less, while higher score became more probable in the treatment arm. The treatment effect *β*_trt_=0.2624=log(1.3) corresponds to an Odds Ratio of OR=1.3.The specific constellation of prognostics factor represented refers to a C8 key muscle, with a Motor Level C5 (*x*_*lev*_=C5.-3), a baseline motor score of *y*_base,*i,m*,1_=1, and an autoregressive component *y*_auto,*i,m*−1,2_=3 for the motor score of the key muscle just above the one being reported

In terms of clinical interpretation of treatment effect estimates, we note that by applying the proposed model, the exponentiated treatment effect estimate $\widehat {\beta _{\text {trt}}}$ can be interpreted as the conditional odds ratio between the treatment and control trial arms, which is a common and accepted way of quantifying treatment effect in the clinical setting. Even when the proportional odds assumption is not fully met, it still provides an interpretable parameter that summarizes the treatment effect over all levels of the outcome [23]. In addition, the transitional model provides motor score probabilities for each combination of prognostic variables, making the direct comparison and visual representation of treated and untreated participants straightforward (see Fig. 3).

On the contrary, clear interpretation of the results produced by common approaches is precluded by summed scores of suppositional metric endpoints, providing little insight for trial scientists and clinicians. Importantly, small and possibly localized treatment effects, which are a hallmark of many neurological disorders, can be disentangled using ordinal approaches for motor scores, but become lost in the analysis of summed total scores.

Finally, our simulation showed (Table 1) that a statistical power of 80 %, which is a common goal for clinical trials planners, is reached by the ordinal model only for large trial size and large postulated treatment effects. As a total trial size of *N* =200 seems to currently represent the practical upper limit for conducting SCI trials, the statistical detection of an existing treatment effect seems to rely on a rather strong effect. Further improvements of the ordinal model will likely result in lowered requirements for treatment detection.

### Revisiting a key SCI trial

To provide a concrete application of our approach, we analysed a subset of participants of the Sygen®; trial [17, 35, 36]. Many ancillary analyses in the original publication were based on t-test and ANCOVA approaches and favoured the treatment group over placebo [17]. In particular, treated participants showed a faster initial recovery than control subjects, who nonetheless caught up at slightly later time points.

On the subsample of patients we considered, no one of the six approaches was significant at the conventional nominal level 0.05. Nonetheless, all approaches showed a tendency towards negative effect of treatment on the UEMS, meaning that treated patient showed on average a slightly worse recovery than patients in the control arm. Especially for the ordinal approach, the results imply that the odds of participants in the treatment group of achieving up to a given motor score were only $\mathrm {e}^{\widehat {\beta _{\text {trt}}}}= 0.82$ times the odds of a participant with similar characteristics in the control arm, indicating a worse recovery for treated patients.

The negative estimate of treatment effect in cervical participants is rather unexpected. The observed unbalance toward more severe lesions in the treatment arm may explain at least in part these results, which nonetheless might be examined more closely to rule out potentially unintended detrimental effects. Nevertheless, we retain that generalizations of our results to the overall validity of the trial and its compound cannot be drawn.

### Are summed overall scores not “good enough” ?

In our application, all six approaches presented delivered comparable results, namely statistically non-significant negative trends for participants in the treatment arm. One may therefore wonder what the added value of an ordinal approach like the proposed transitional ordinal model is. Briefly, routinely employed approaches based on summed overall scores imply:

- Unmet assumptions: adding ordinal endpoints to form a single overall score requires equal differences across all ordinal scales as well as unidimensionality. Both assumptions are usually not further investigated [37], but the first can be rejected on medical reasons only, while the latter does not hold for several SCI endpoints (e.g. FIM [39], SCIM [40]).
- Flawed inference and estimation: known practical consequences of assuming metric property for ordinal endpoints are biased parameter estimates and misleading associations [21–23].
- Reduced statistical power: small and possibly localised effects are expected to be the hallmark of spinal cord injury rehabilitation strategies. The simulation reported provide evidence for a much lower capacity of approaches based on summed scores to detect existing treatment effects. Lower power also translates in higher requirement for trial participants.
- Unclear interpretation of treatment effect: a clear interpretation of treatment effect estimates as conditional OR, which can be visualised for each key muscle separately (see Fig. 3), is not possible for summed scores.
- Limited future extensions: future refinement of routinely employed approaches are strongly limited by the underlying, inappropriate analysis approach. Instead, ordinal approaches, which are based on a regression framework, easily accommodates for extensions (e.g. further prognostic factors, interactions, localised effects).

Concluding, from a theoretical point of view, routinely employed approaches have little scientific validity and have been replaced by more rigorous approaches. Even more importantly, they are also potentially misleading on practical terms. Our flexible model represents therefore an improved and pragmatic solution to the analysis of this type of complex ordinal endpoints.

### Brain Injury: similar issues, similar solutions

We observe that most of the discussion points we raised link to the report by the International Mission on Prognosis and Clinical Trial Design in Traumatic Brain Injury TBI [45]. TBI is a related clinical field which faced very similar challenges, mainly related to the heterogeneity of the patient population, and had a similar history of clinical testing as SCI.

In fact, TBI also experienced a disappointing progression of clinical testing of treatment interventions in spite of extremely promising pre-clinical data and early phase trials. Maas et al. [45] reported that a key difficulty has been the inherent heterogeneity TBI subjects, and that the observed development was due, at least to some extent, to limitations in the trial designs and analyses. Both aspects have also been reported as hallmarks of SCI research.

Summarizing, The TBI Mission solicited the TBI community to [45]:

- provide details of the major baseline prognostic characteristics
- broaden inclusion criteria as much as is it compatible with the current understanding of the mechanisms of action of the intervention
- incorporate pre-specified covariate adjustment into the statistical analysis
- use an ordinal approach for the statistical analysis

A part from the first recommendation, which is mainly concerned with the way clinical studies are reported, the following three points regard the planning and especially the analysis of clinical trials in TBI, and are implemented in this publication. Selection of patients is based only on the initial Motor Level, which relates to the understanding of motor function. The proposed model (see Eq. 2) both include the most relevant covariates adjustment, namely baseline motor scores as well as motor lesion, and uses and ordinal approach for ordinal data based on the proportional odds model.

### Latent variable models: an improved, readily available framework

More generally speaking, the statistical foundations of regression models for ordinal endpoints were developed more than 4 decades ago [46–48], and have ever since undergone a steady development. There is a huge body of literature pertaining to the analysis of ordinal variables, including Item Response Theory IRT and mixed-effects models for ordinal variables [49]. Despite this development, most clinical trials in neurology still rely on surpassed approaches [44], corroborating the negative trend of methodological errors related to the analysis of ordinal scales in medical research [50].

The proposed transitional ordinal model (Eq. 2) is an extension of the well known proportional odds model (e.g. [51]). The latter can be seen as an important special case within the IRT framework, and is closely related to the Rasch model [46]. All these statistical models are generally referred to as latent variable models, because they find application in situations where a set of ordinal variables are seen as indicators of a latent variable. This latent variable is the main interest of the analysis, and, although it cannot be measured directly, it can be inferred from the available ordinal variables. The latent variable approach seems both appropriate and appealing for applications in the clinical setting, and the transitional ordinal model proposed draws a concrete link from SCI to latent variable models. Further extensions of our approach can be tailored to the analysis of other endpoints such as functional assessments and PROs. In fact, the analysis of PRO, and the related trial powering based on Rasch models has recently received much attention [52, 53]. We believe that the transition from currently employed analysis approaches to more sophisticated models within the readily available framework of latent variable models would represent a great scientific progression for the planning and analysis of complex neurological endpoints.

## Conclusion

We propose an autoregressive transitional ordinal model for the analysis of a specific SCI endpoint which takes into account the complex ordinal nature of the endpoint under investigation and explicitly accounts for relevant prognostic factors. Superior statistical power in virtually all settings, combined with a clear clinical interpretation of treatment effect and widespread availability on commercial softwares, are strong arguments for clinicians and trial scientists to adopt, and further refine, the proposed approach.

## Additional file

Additional file 1 International Standards for Neurological Classification of Spinal Cord Injury. (PDF 935 kb)

## Abbreviations

EMSCI: European multicenter study about spinal cord injury
FIM: Functional independence measure
ICCP: International campaign for cures of spinal cord injury paralysis
IRT: Item response theory ISNCSCI: Int. standards for neurological classification of spinal cord injury
ML: Motor level
OR: Odds ratio
PRO: Patient reported outcomes
RCT: Randomized clinical trial
SCI: Spinal cord injury
SCIM: Spinal cord independece measure
TBI: Traumatic brain injury
UEMS: Upper Extremity Motor Scores
