# Supplementary material for: Autoregressive transitional ordinal model to test for treatment effect in neurological trials with complex endpoints
Source: BMC Med Res Methodol. 2016 Nov 8;16:149. doi: 10.1186/s12874-016-0251-y (PMC5100172; doi:10.1186/s12874-016-0251-y)
Supplement: Additional file 1 — International Standards for Neurological Classification of Spinal Cord Injury. (PDF 935 kb) [file 12874_2016_251_MOESM1_ESM.pdf]

AMERICAN SPINAL INJURY ASSOCIATION

INTERNATIONAL STANDARDS FOR NEUROLOGICAL CLASSIFICATION OF SPINAL CORD INJURY (ISNCSCI)

ISCOS INTERNATIONAL SPINAL CORD SOCIETY

Patient Name______________________ Date/Time of Exam ______________________

Examiner Name ______________________ Signature ______________________

RIGHT

	MOTOR KEY MUSCLES		SENSORY KEY SENSORY POINTS Light Touch (LTR)	Pin Prick (PPR)
		C2		
		C3		
		C4		
UER (Upper Extremity Right)	*Elbow flexors*	C5		
	Wrist extensors	C6		
	Elbow extensors	C7		
	Finger flexors	C8		
	Finger abductors (little finger)	T1		
		T2		
		T3		
		T4		
		T5		
		T6		
		T7		
		T8		
		T9		
		T10		
		T11		
		T12		
		L1		
LER (Lower Extremity Right)	*Hip flexors*	L2		
	Knee extensors	L3		
	Ankle dorsiflexors	L4		
	Long toe extensors	L5		
	Ankle plantar flexors	S1		
		S2		
		S3		
(VAC) Voluntary anal contraction (Yes/No)		S4-5		
RIGHT TOTALS (MAXIMUM)	(50)		(56)	(56)

Comments *(Non-key Muscle? Reason for NT? Pain?):*

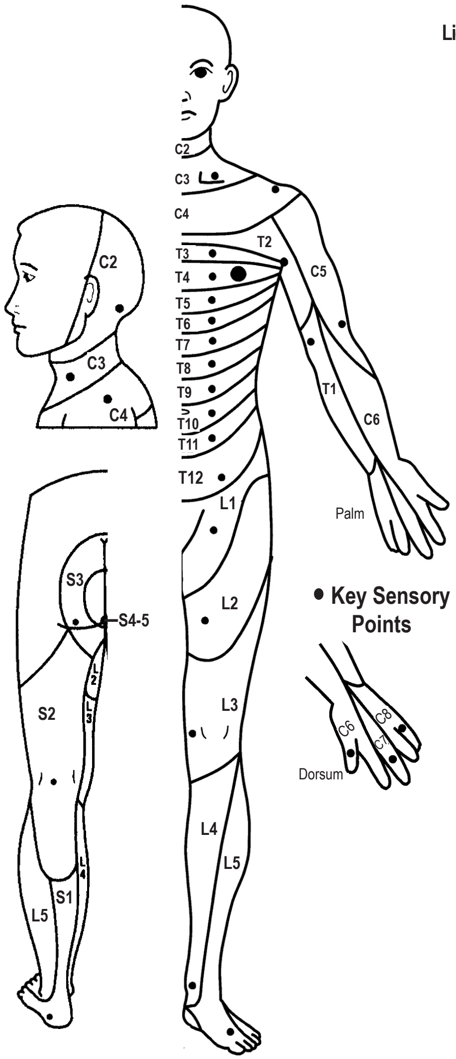

LEFT

SENSORY KEY SENSORY POINTS Light Touch (LTL)	Pin Prick (PPL)		MOTOR KEY MUSCLES		
		C2			
		C3			
		C4			
		C5		*Elbow flexors*	UEL (Upper Extremity Left)
		C6		*Wrist extensors*	
		C7		*Elbow extensors*	
		C8		*Finger flexors*	
		T1		*Finger abductors (little finger)*	
		T2			
		T3			
		T4			
		T5			
		T6			
		T7			
		T8			
		T9			
		T10			
		T11			
		T12			
		L1			
		L2		*Hip flexors*	LEL (Lower Extremity Left)
		L3		*Knee extensors*	
		L4		*Ankle dorsiflexors*	
		L5		*Long toe extensors*	
		S1		*Ankle plantar flexors*	
		S2			
		S3			
		S4-5	***(DAP) Deep anal pressure (Yes/No)***		
(56)	(56)		(50)	***LEFT TOTALS*** (MAXIMUM)	

MOTOR
(SCORING ON REVERSE SIDE)

0 = total paralysis
1 = palpable or visible contraction
2 = active movement, gravity eliminated
3 = active movement, against gravity
4 = active movement, against some resistance
5 = active movement, against full resistance
5 = normal corrected for pain/disuse*
NT = not testable

SENSORY
(SCORING ON REVERSE SIDE)

0 = absent *2 = normal*
1= altered *NT = not testable*

MOTOR SUBSCORES

UER ____ + UEL ____ = ***UEMS TOTAL*** ____
MAX (25) *(25)* *(50)*

LER ____ + LEL ____ = ***LEMS TOTAL*** ____
MAX (25) *(25)* *(50)*

SENSORY SUBSCORES

LTR ____ + LTL ____ = ***LT TOTAL*** ____
MAX (56) *(56)* *(112)*

PPR ____ + PPL ____ = ***PP TOTAL*** ____
MAX (56) *(56)* *(112)*

NEUROLOGICAL LEVELS
Steps 1-5 for classification as on reverse

	R	L
1. SENSORY		
2. MOTOR		

3. NEUROLOGICAL LEVEL OF INJURY (NLI) ____

4. COMPLETE OR INCOMPLETE? ____
Incomplete = Any sensory or motor function in S4-5

5. ASIA IMPAIRMENT SCALE (AIS) ____

(In complete injuries only)
ZONE OF PARTIAL PRESERVATION
Most caudal level with any innervation

	R	L
SENSORY		
MOTOR		

This form may be copied freely but should not be altered without permission from the American Spinal Injury Association.

Muscle Function Grading

0 = total paralysis

1 = palpable or visible contraction

2 = active movement, full range of motion (ROM) with gravity eliminated

3 = active movement, full ROM against gravity

4 = active movement, full ROM against gravity and moderate resistance in a muscle specific position

5 = (normal) active movement, full ROM against gravity and full resistance in a functional muscle position expected from an otherwise unimpaired person

5* = (normal) active movement, full ROM against gravity and sufficient resistance to be considered normal if identified inhibiting factors (i.e. pain, disuse) were not present

NT = not testable (i.e. due to immobilization, severe pain such that the patient cannot be graded, amputation of limb, or contracture of > 50% of the normal range of motion)

Sensory Grading

0 = Absent

1 = Altered, either decreased/impaired sensation or hypersensitivity

2 = Normal

NT = Not testable

Non Key Muscle Functions (optional)

May be used to assign a motor level to differentiate AIS B vs. C

Movement	Root level
**Shoulder:** Flexion, extension, abduction, adduction, internal and external rotation<br>**Elbow:** Supination	**C5**
**Elbow:** Pronation<br>**Wrist:** Flexion	**C6**
**Finger:** Flexion at proximal joint, extension.<br>**Thumb:** Flexion, extension and abduction in plane of thumb	**C7**
**Finger:** Flexion at MCP joint<br>**Thumb:** Opposition, adduction and abduction perpendicular to palm	**C8**
**Finger:** Abduction of the index finger	**T1**
**Hip:** Adduction	**L2**
**Hip**: External rotation	**L3**
**Hip:** Extension, abduction, internal rotation<br>**Knee:** Flexion<br>**Ankle:** Inversion and eversion<br>**Toe:** MP and IP extension	**L4**
**Hallux and Toe:** DIP and PIP flexion and abduction	**L5**
**Hallux:** Adduction	**S1**

ASIA Impairment Scale (AIS)

A = Complete. No sensory or motor function is preserved in the sacral segments S4-5.

B = Sensory Incomplete. Sensory but not motor function is preserved below the neurological level and includes the sacral segments S4-5 (light touch or pin prick at S4-5 or deep anal pressure) AND no motor function is preserved more than three levels below the motor level on either side of the body.

C = Motor Incomplete. Motor function is preserved below the neurological level**, and more than half of key muscle functions below the neurological level of injury (NLI) have a muscle grade less than 3 (Grades 0-2).

D = Motor Incomplete. Motor function is preserved below the neurological level**, and at least half (half or more) of key muscle functions below the NLI have a muscle grade $\geq$ 3.

E = Normal. If sensation and motor function as tested with the ISNCSCI are graded as normal in all segments, and the patient had prior deficits, then the AIS grade is E. Someone without an initial SCI does not receive an AIS grade.

** For an individual to receive a grade of C or D, i.e. motor incomplete status, they must have either (1) voluntary anal sphincter contraction or (2) sacral sensory sparing with sparing of motor function more than three levels below the motor level for that side of the body. The International Standards at this time allows even non-key muscle function more than 3 levels below the motor level to be used in determining motor incomplete status (AIS B versus C).

NOTE: When assessing the extent of motor sparing below the level for distinguishing between AIS B and C, the ***motor level*** on each side is used; whereas to differentiate between AIS C and D (based on proportion of key muscle functions with strength grade 3 or greater) the ***neurological level of injury*** is used.

AMERICAN SPINAL INJURY ASSOCIATION

INTERNATIONAL STANDARDS FOR NEUROLOGICAL CLASSIFICATION OF SPINAL CORD INJURY

ISCOS

INTERNATIONAL SPINAL CORD SOCIETY

Steps in Classification

The following order is recommended for determining the classification of individuals with SCI.

1. Determine sensory levels for right and left sides.
The sensory level is the most caudal, intact dermatome for both pin prick and light touch sensation.

2. Determine motor levels for right and left sides.
Defined by the lowest key muscle function that has a grade of at least 3 (on supine testing), providing the key muscle functions represented by segments above that level are judged to be intact (graded as a 5).
Note: in regions where there is no myotome to test, the motor level is presumed to be the same as the sensory level, if testable motor function above that level is also normal.

3. Determine the neurological level of injury (NLI)
This refers to the most caudal segment of the cord with intact sensation and antigravity (3 or more) muscle function strength, provided that there is normal (intact) sensory and motor function rostrally respectively.
The NLI is the most cephalad of the sensory and motor levels determined in steps 1 and 2.

4. Determine whether the injury is Complete or Incomplete.
(i.e. absence or presence of sacral sparing)
*If voluntary anal contraction = **No** AND all S4-5 sensory scores = **0** AND deep anal pressure = **No,** then injury is **Complete.***
*Otherwise, injury is **Incomplete.***

5. Determine ASIA Impairment Scale (AIS) Grade:

Is injury Complete? **If YES, AIS=A** and can record ZPP (lowest dermatome or myotome on each side with some preservation)

NO ↓

Is injury Motor Complete? **If YES, AIS=B**

NO ↓ (No=voluntary anal contraction OR motor function more than three levels below the motor level on a given side, if the patient has sensory incomplete classification)

Are at least half (half or more) of the key muscles below the neurological level of injury graded 3 or better?

NO ↓ **AIS=C**

YES ↓ **AIS=D**

If sensation and motor function is normal in all segments, AIS=E
Note: AIS E is used in follow-up testing when an individual with a documented SCI has recovered normal function. If at initial testing no deficits are found, the individual is neurologically intact; the ASIA Impairment Scale does not apply.
